# Similar Motor Block Effects and Disposition Kinetics between Lidocaine and (±)Mepivacaine in Patients Undergoing Axillary Brachial Plexus Block during Day Case Surgery

**DOI:** 10.1100/tsw.2002.180

**Published:** 2002-05-14

**Authors:** Marc A. M. Simon, Tom B. Vree, Mathieu J.M. Gielen, Leo H.D.J. Booij, Aart J. Lagerwerf

**Affiliations:** Department of Anaesthesiology, Medisch Spectrum Twente, Haaksbergerstraat 55, 7513 ER Enschede, Netherlands; Institute for Anaesthesiology, Academic Hospital Nijmegen Sint Radboud, Geert Grooteplein Zuid 10, 6525 GA Nijmegen, Netherlands

**Keywords:** lidocaine, mepivacaine, regional analgesia, elimination kinetics, disposition

## Abstract

The aim of this investigation was to compare the clinical effects and pharmacokinetics of lidocaine (one metabolite) and mepivacaine (two metabolites) in 2 groups of 15 patients undergoing axillary brachial plexus anaesthesia. The study had a randomised design. The 30 patients were divided into 2 groups. The patients received either lidocaine (600 mg = 2.561 mMol + 5 μg ml^-1^ adrenaline) or mepivacaine (600 mg = 2.436 mMol + 5 μg ml^-1^ adrenaline), injected via the axilla near the brachial plexus over a period of 30 s. Onset of surgical analgesia was defined as the period from the end of the local anaesthetic injection to the loss of pinprick sensation in the distribution of the ulnar, radial, and median nerve. Motor block was measured. Onset of motor block was similar for both drugs. Lidocaine is eliminated biexponentially with a t_1/2α_ of 9.95 ± 14.3 min and a t_1/2β_ of 2.86 α 1.55 h. Lidocaine is metabolised into MEGX (t_max_ 2.31 α 0.84 h; Cmax 0.32 α 0.13 mg l^-1^; t_1/2β_ 2.36 α 2.35 h; total body clearance was 67.9 α 28.9 l h^-1^).

Mepivacaine is eliminated rapidly and monoexponentially with a t_1/2_ of 4.78 α 2.38 h, a C_max_ of 3.89 α 0.83 mg l^-1^, and a t_max_ of 0.41 α 0.19 h. The total body clearance of mepivacaine is 50% of that of lidocaine, 26.9 α 10.6 l h^-1^ vs. 67.9 α 28.9 l h^-1^, respectively (*p* <0.0001). (α)Mepivacaine is metabolised into (α)4-OHmepivacaine (C_max_ 0.45 α 0.25 mg l^-1^; t_1/2β_ 6.48 α 6.57 h) and (α)2,6-pipecoloxylidide (C_max_ 0.56 α 0.30 mg l^-1^; t_1/2b_ 1.48 α 0.74 h).

For the axillary brachial plexus block, lidocaine and mepivacaine show similar pharmacodynamic and pharmacokinetic behaviour, despite the number of metabolites, and can therefore be used to the clinical preference for this regional anaesthetic technique.

### INTRODUCTION

For (short-lasting) surgical procedures on the hand and forearm, intravenous regional anaesthesia (IVRA)[1] and axillary brachial plexus block are popular techniques[2,3,4].

With the IVRA technique the local anaesthetic drug is administered intravenously in the bloodless arm, while with the brachial plexus block, the drug is administered into the neurovascular sheath. If surgery demands analgesia of the whole arm, the local anaesthetic must be administered around the brachial plexus in the axilla, or even more proximally. When the drug is administered into the neurovascular sheath, it diffuses into the nerves but also into the adjacent vessels and enters the systemic circulation[4]. After brachial plexus disposition of the local anaesthetic, a pharmacokinetic curve will result, which resembles an oral or intramuscular drug concentration time curve. This means there must be a clear absorption phase.

Good surgical analgesia is achieved with most local anaesthetic agents, but onset and recovery will be different. An applied local anaesthetic technique is safe, provided a suitable local anaesthetic agent is properly used. Lidocaine and mepivacaine are considered to be suitable agents for axillary brachial plexus blockade[3,5,6,7,8,9,10,11]. With day case surgery it is considered to be safe for patients to leave the hospital free of anaesthetic drugs[12,13].

Relatively little has been published on the pharmacokinetics of lidocaine and mepivacaine with their metabolites when used for axillary brachial plexus anaesthesia. Both compounds are eliminated by hydrolysis of the amide bond and by oxidation. Both compounds form a measurable metabolite in plasma, i.e., (±)4´-hydroxymepivacaine with (±)2,6-pipecoloxylidide (PPX) from (±)mepivacaine and MEGX (MonoEthylGlycylXylidide) from lidocaine, respectively. The aim of this investigation is to compare the clinical effect and the pharmacokinetics of lidocaine and (±)mepivacaine with their metabolites in 2 groups of 15 patients undergoing brachial plexus anaesthesia by axillary disposition.

### MATERIALS AND METHODS

#### Patients

The hospital ethics committee of the Medisch Spectrum Twente approved the study, and written consent to participate was obtained from 15 patients scheduled for hand or forearm surgery (Table 1). All patients were classified according to the criteria of the American Society of Anesthesiologists as ASA I or II. Seven men and eight women received mepivacaine. The mean (±s.d.) body weight was 73.5 ± 13.5 kg, body length was 1.74 ± 0.06 m, and age was 49.7 ± 12.3 years.

Ten men and five women received lidocaine. The mean (±s.d.) body weight was 80.4 ± 11.7 kg, body length was 1.73 ± 0.06 m, and age was 55.5 ± 14.2 years. The study had a double-blind randomised design.

#### Patient Preparation

No premedication was given. Two cannulae were introduced into the arm that was not receiving surgery; one into a suitable vein and the other into the radial artery. The latter was used for continuous monitoring of the arterial blood pressure and for intermittent blood sampling.

Oxygen saturation, pulse rate and EKG, and arterial blood pressure were measured continuously via a Datex “Satlite,” (Datex Division of Instrumentarium Corporation, Helsinki, Finland), from the first venous cannulation until withdrawal of the final arterial blood sample.

Axillary block was performed with a Stimuplex 50-mm short-bevel needle connected to the Stimuplex nerve stimulator (B. Braun Melsungen AG, Melsungen, Germany). Using a stimulation mode of 0.5 mA, 0.3 ms, and 1.0 Hz, perivascular puncture was performed until muscle contrac-

**TABLE 1**
**Type of Surgery in Both Groups of Patients**

| Patient No. | *Lidocaine* Surgery | *Mepivacaine* Surgery |
|---|---|---|
| 1 | Dupuytren | Tendon suture digit 2 |
| 2 | Neurome palm of hand | Dupuytren |
| 3 | Arthroplasty saddle joint | Dupuytren |
| 4 | Nerve transplantation right arm | Tendon suture digit V |
| 5 | Dupuytren | Dupuytren |
| 6 | Nerve transplantation left arm | Arthrodese DIP digit III |
| 7 | Tenolysis left underarm | Correction ulnaris deviation |
| 8 | Synovectomy left wrist dorsal | Carpal tunnel |
| 9 | Dupuytren | Arthroscopy right wrist |
| 10 | Carpal tunnel | Dupuytren |
| 11 | Neurolysis N Medianus left wrist | Dupuytren |
| 12 | Dupuytren | Tendon suture digit 2 |
| 13 | Distal ulnar resection | Dupuytren |
| 14 | Arthrodese DIP digit V | Arthroplasty saddle joint digit 1 |
| 15 | Dupuytren | Plastic saddle joint digit 1 |

tions indicating stimulation of the median nerve were observed. Over a period of about 30 s, 40 ml of the local anaesthetic solution were then injected. Concurrently, pressure was applied with the palpating fingers to the neurovascular sheath just distal to the point of entry of the needle.

Completion of local anaesthetic injection was designated t = 0. Sensory block development was measured by loss of sensation to pinprick in the cutaneous distributions of the sensory nerves innervating the hand, namely the median nerve, the radial nerve, and the ulnar nerve. Sensory block was graded according to Hollmén[9] on a three-point rating scale (1 = absence of cold sensation, 2 = analgesia, 3 = anaesthesia). Sensory block testing commenced at t = 0 and was continued at 2-min intervals for the first 20 min and at 5-min intervals for the next 20 min. When a sensory block 3 was not achieved within 40 min, additional systemic analgesics were administered or infiltration with 0.5% bupivacaine in the surgical field was given.

Onset of the surgical analgesia was defined as the period from the end of the injection of the local anaesthetic to a loss of pinprick sensation (a sensory block 3 score) in the distribution of all three nerves.

Motor block was assessed by the Baseline Hydraulic Hand Dynamometer (Fabrication Enterprises, Inc., Irvington, NY). This device measures the squeeze force, i.e., muscle strength, of the hand and forearm in either kilograms or pounds. A normal baseline value was established before commencing the axillary block and designated 100%. The first assessment for motor block was performed 2 min after the end of the local anaesthetic injection (t = 2) and then repeated at a 2-min interval for the first 20 min, and at 5-min intervals thereafter in those patients who had not yet achieved a zero value (100% blockade). The percentage decline in muscle strength from the base line could then be calculated.

### Drugs

Lidocaine (1.5%) (Xylocaine®, +5 $\mu g\ l^{-1}$ adrenaline ) and mepivacaine (1.5%) (Scandicaine®, +5 $\mu g\ l^{-1}$ adrenaline) were obtained from Astra Pharmaceuticals (Rijswijk, the Netherlands).

Over a period of 30 s, 40 ml of the lidocaine solution ($40 \times 15$ mg $ml^{-1}$ = 600 mg = 2.561 mMol) were injected around the brachial plexus to each of the patients in the lidocaine group.

Additionally, 40 ml of the mepivacaine solution ($40 \times 15$ mg $ml^{-1}$ = 600 mg = 2.436 mMol) were injected around the brachial plexus over a period of 30 s to each of the patients in the mepivacaine group.

### Side Effects

Any skin reactions or subjective complaints were noted.

### Sampling

A total of 18 arterial blood samples were taken from each patient. One was drawn immediately before injection (t = 0) and subsequent samples 5, 10, 15, 20, 25, 30, 40, 50, 60, 75, 90, 105, 120, 135, 150, 165, and 180 min. Blood was collected in tubes containing Li-heparin. The samples were centrifuged at 3,000 g, and the plasma separated and stored at –20°C until analysis.

### Analysis

The plasma concentrations of (±)mepivacaine ($C_{15}H_{22}N_2O$; CAS number 96-88-8; MW 246.35; HCl salt CAS number 1722-62-9, MW 282.9) with metabolites (±)4´-hydroxymepivacaine ($C_{15}H_{22}N_2O_2$) and (±)2,6-pipecoloxyilidide (PPX) were determined by a modified HPLC method as described earlier[14]. Briefly, the method is as follows: Column: Spherisorb 5 ODS, $250 \times 4.6$ mm. UV detection was achieved at 210 nm. Mobile phase: (1 g $H_3PO_4$, 0.45 g TMACl in 1 l distilled water) and acetonitrile (77.5:22.5, v/v) at 1.0 ml $min^{-1}$ flow rate. Plasma (0.2 ml) was deproteinised with 0.3 ml acetonitrile, vortexed, and centrifuged at 3,000 g. 50 ml was injected onto the column. Retention times were: 4-OHmepivacaine 5.6 min, PPX 9.6 min, mepivacaine 13.2 min. The limit of quantification was 0.30 μg $ml^{-1}$ for both compounds. The inter- and intraday coefficients of variance for mepivacaine (0.3 –5.0 μg $ml^{-1}$) and both metabolites (0.3–1.0 μg $ml^{-1}$) were less than 5%.

The plasma concentration of lidocaine ($C_{14}H_{22}N_2O$; CAS number 137-58-6; MW 234.33; pKa 7.9; HCL.$H_2O$ salt CAS number 73-78-9; MW 288.8) and its metabolite MEGX monoethylglycylxylidide ($C_{12}H_{17}N_2O$; MW 220.33) were determined by the same method. The inter- and intraday coefficients of variance for lidocaine and the metabolite were less than 5%.

### Pharmacokinetics

Pharmacokinetic parameters were calculated using a two-compartment model for lidocaine, a one-compartment model for mepivacaine and its two metabolites, and a noncompartimental analysis using the MW/Pharm computer package (Mediware[R], Groningen, the Netherlands)[15].

$C_{max}$, the maximum plasma concentration (mg $l^{-1}$) read from the fitted plasma concentration–time curve ($r^2 > 0.98$); $t_{max,}$ the time (h) at which $C_{max}$ occurred; the elimination half-life associated with the terminal slope of a semilogarithmic concentration–time curve (ln2/λ, [h]), where λ = elimination constant; $AUC_t$, $AUC_\infty$, the area under the plasma concentration–time curve (mg h $l^{-1}$) calculated (linear trapezoidal method), until the last measured concentration ($C_t$) or extrapolated to $C_t$ infinity, respectively; $t_{1/2absorption}$, the absorption half-life (h); $t_{1/2\alpha}$, the half-life of the fast elimination phase, and $t_{1/2\beta}$ that of the terminal elimination phase; $CL_t$, body clearance is F. Dose/$AUC_t$ (assuming F = 1).

CL, total body clearance is F. Dose/$AUC_\infty$, assuming the bioavailability F = 1); $V_d$ = F. Dose/$C_o$, the volume of distribution in the central compartment (F = 1); $V_\beta$ = CL/β, the volume of distribution in the second compartment. $V_{ss}$ = F. Dose . $AUMC_\infty/AUC_\infty^2$, the volume of distribution at steady state (F = 1). Mean residence time (MRT) = $AUMC_\infty/AUC_\infty$, where $AUMC_\infty$ is the area under the moment curve from zero to infinity.

### Statistical Analysis

The Mann-Whitney two-tailed test for independent (unpaired) observations was used. Statistical significance was defined as $p < 0.05$.

## RESULTS

### Clinical Response

Onset time of sensory block of the median nerve of both drugs was similar. In 27 patients, satisfactory surgical conditions were reached, as evidenced by good sensory blockade. None of the patients showed objective symptoms of toxicity, either local or systemic, during and after injection of the local anaesthetic, nor were there any subjective complaints. Clinically insignificant changes in blood pressure, heart rate and rhythm, or oxygen saturation were observed at any time during the procedure.

Two patients in the lidocaine group failed to develop sensory block in the distribution of the radial nerve within an hour after injection. In both individuals infiltration of the surgical area was necessary with 5 ml bupivacaine 0.5% (Astra). A third patient in the same group failed to develop any sensory block whatsoever and was given general anaesthesia. The remaining 12 individuals achieved good surgical anaesthesia without additional systemic analgesics.

Decline in muscle power, expressed as percentage of the preanaesthetic baseline, is plotted vs. time in Fig. 1A,B. All patients developed a complete motor block within 20 min of injection. Four patients in the lidocaine group and five patients in the mepivacaine group showed a complete motor block within 2 min, and one patient in each group needed 20 min to reach the 100% motor block. The remaining patients reached full blockade in varying periods of time as shown in Fig. 1A,B.

The mean lidocaine motorblock–time-effect curve showed two phases, characterised by a $t_{1/2\alpha}$ of 0.098 min and a $t_{1/2\beta}$ of 4.0 min. Mepivacaine showed a similar effect, and the mean motor block-time effect curves of lidocaine and mepivacaine were similar ($p$ = NS).

### Pharmacokinetics

#### *Lidocaine*

Figure 2 and Table 2 show the mean plasma concentration–time curves of lidocaine and its metabolite MEGX in 15 patients after axillary administration of the local anaesthetic drug. Lidocaine was quickly absorbed from the tissues ($t_{1/2abs}$ = 0.14 ± 0.05 h), resulting in a low $t_{max}$ value of 0.43 ± 0.19 h and a $C_{max}$ of 2.87 ± 1.19 mg $l^{-1}$. The metabolic formation of MEGX by N-dealkylation started immediately after administration, and the plasma concentration grew in 3 h to 17% of that of the parent compound. The elimination of lidocaine is biexponential with a short $t_{1/2\alpha}$ of 9.95 ± 14.3 min and a $t_{1/2\beta}$ of 2.86 ± 1.55 h (Table 3).

The formation and elimination of the metabolite MEGX can be described with a one-compartment model. The apparent $t_{1/2absorption}$ of MEGC (reflecting its rate of formation minus elimination) is 0.70 ± 0.43 h, which is five times longer than that of lidocaine ($p < 0.0001$). The $t_{max}$ (2.31 ± 0.84 h) of MEGX is also five times longer than that of lidocaine ($p < 0.0001$), while its $C_{max}$ is lower (0.32 ± 0.12 mg $l^{-1}$, $p < 0.0001$). The elimination half life of MEGX is 2.36 ± 2.35 h and is similar to that of lidocaine ($p = 0.49$, Table 4).

#### *Mepivacaine*

Figure 3 shows the mean plasma concentration–time curves of mepivacaine and its metabolites 4-OHmepivacaine and 2,6-pipecoloxylidide (PPX) in 15 patients after axillary administration of the

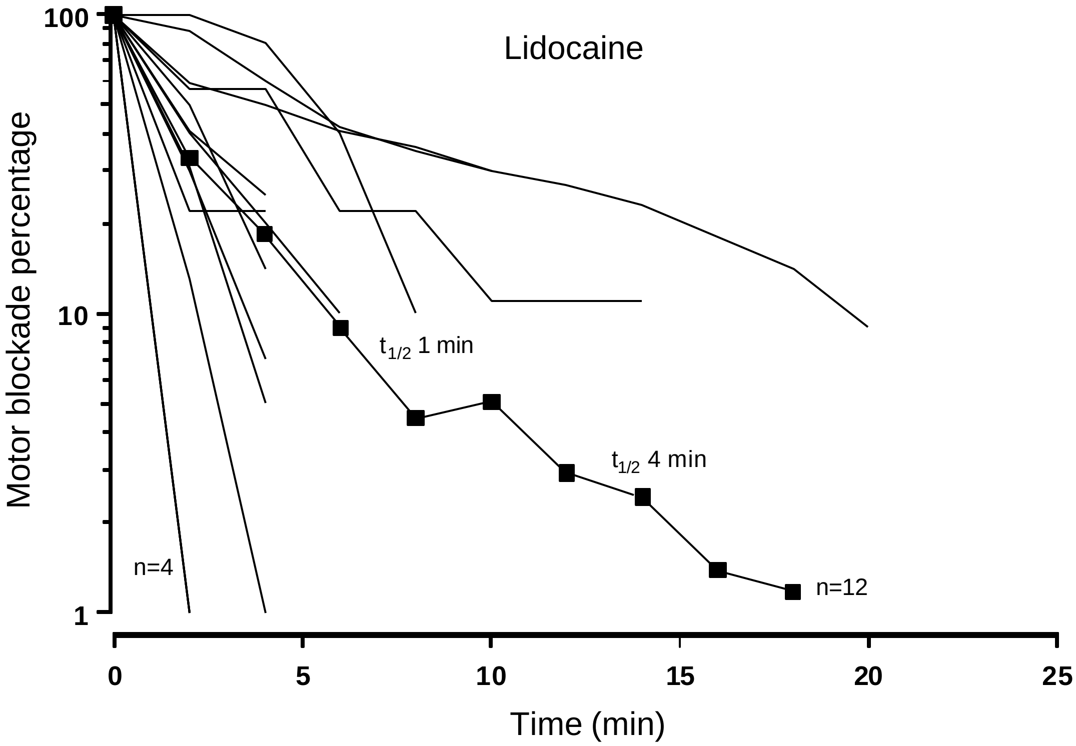

**FIGURE 1A**

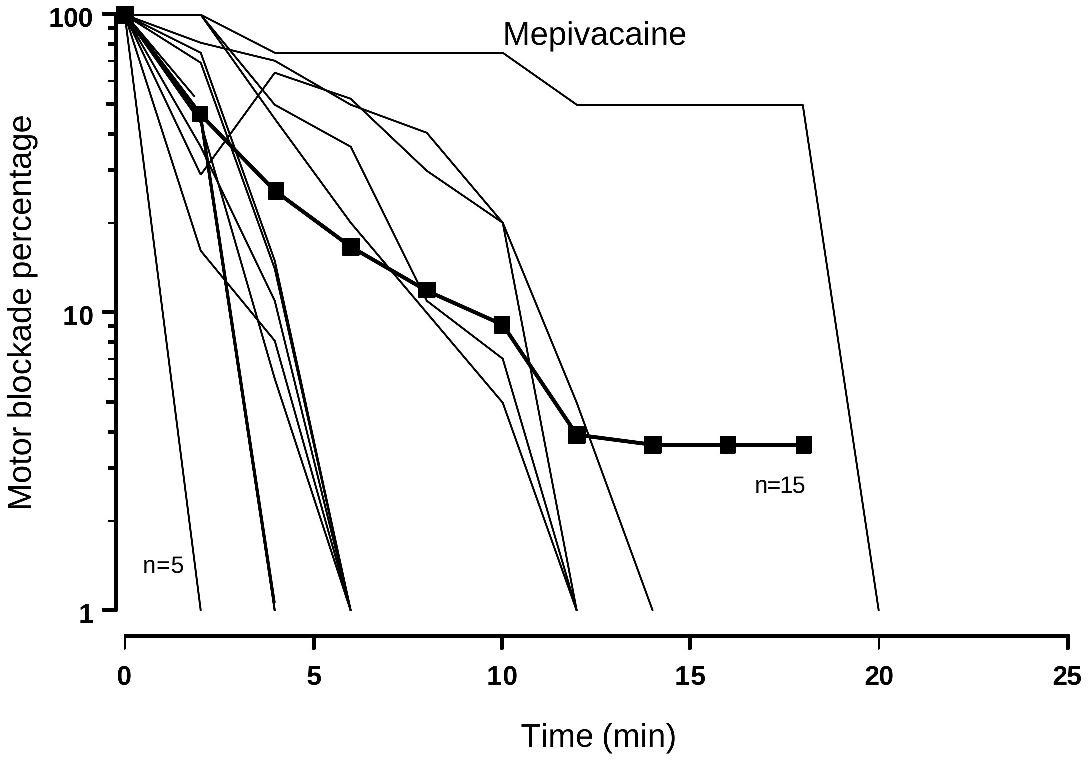

**FIGURE 1B**

**FIGURE 1**. (A) Individual and mean (n = 12) percentage motorblockade–time curves of lidocaine, after axillary administration of 600 mg lidocaine (+ 5 µg ml$^{-1}$ adrenaline). Three patients failed to develop a motor block. (B) Individual and mean (n = 15) percentage motorblockade–time curves of mepivacaine, after axillary administration of 600 mg mepivacaine (+ 5 µg ml$^{-1}$ adrenaline).

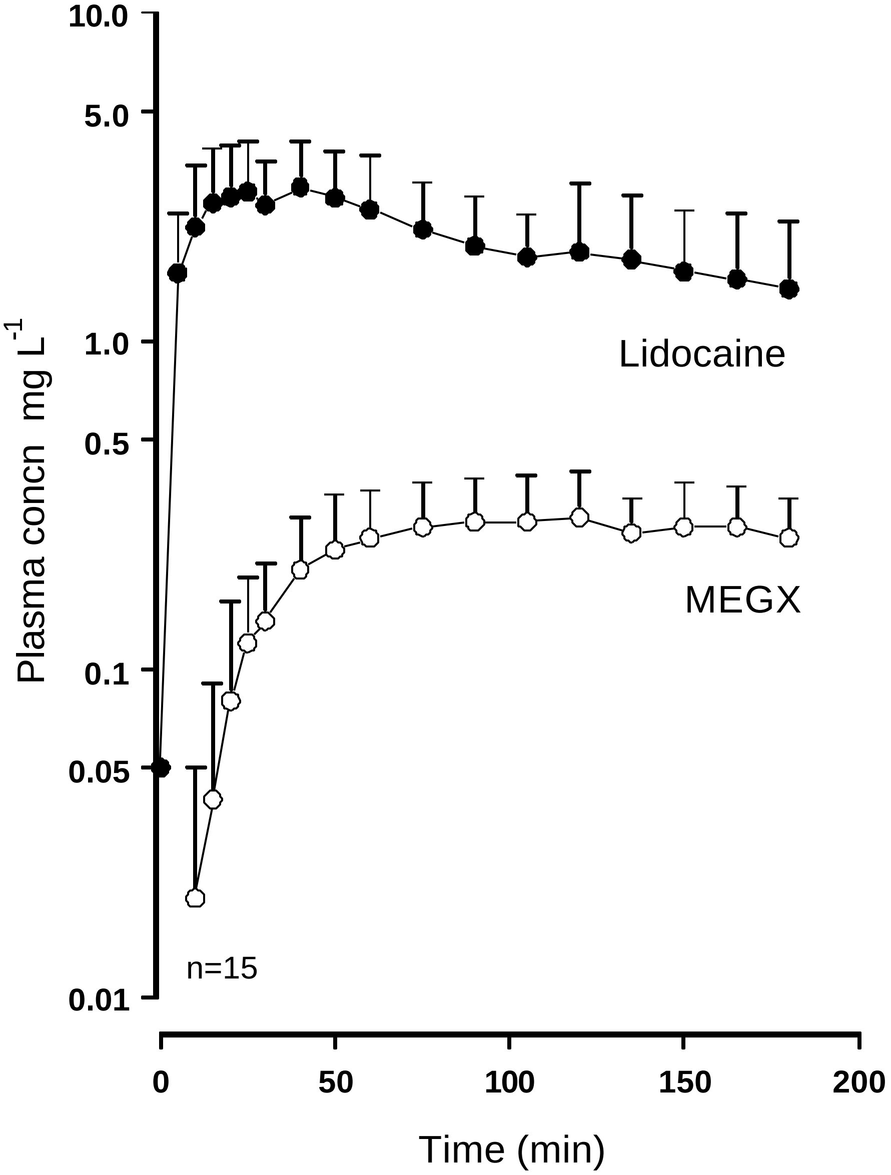

**FIGURE 2.** Mean plasma concentration–time curves of lidocaine (mg $l^{-1}$ ± s.d.), its metabolite MEGX after axillary administration of 600 mg lidocaine (+ 5 µg $ml^{-1}$ adrenaline) (n = 15).

local anaesthetic drug. Mepivacaine was quickly absorbed from the tissues, resulting in a low $t_{max}$ value of 0.41 ± 0.19 h, which was found almost similar to that of lidocaine ($p$ = 0.32), and a $C_{max}$ of 3.89 ± 0.83 mg $l^{-1}$, which was higher than that of lidocaine ($p$ = 0.0157). Mepivacaine is oxidised into 4´-hydroxymepivacaine, and N-dealkylated into 2,6-pipecoloxylidide (PPX). The elimination of mepivacaine is monoexponential, with a $t_{1/2b}$ of 4.78 ± 2.38 h.

Table 3 shows the mean values (± s.d.) and statistical differences in the pharmacokinetic parameters of lidocaine and mepivacaine. The total body clearance of lidocaine is three times higher than that of mepivacaine, 67.9 ± 28.9 l $h^{-1}$ vs. 26.9 ± 10.6 l $h^{-1}$, respectively ($p$ < 0.0008).

**TABLE 2**
**Mean Plasma Concentrations (mg $l^{-1}$ ± s.d.) of Mepivacaine with Metabolite 4-OH and PPX and Lidocaine with its Metabolite MEGX after Axillary Administration of 40 ml 1.5% = 600 mg (+ 5 μg $ml^{-1}$ Adrenaline) (n = 15)**

| Time (min) | *Lidocaine* | *MEGX* | *%** | *Mepivacaine* | *4-OH* | *PPX* |
|---|---|---|---|---|---|---|
| 5 | 1.61 ± 0.82 | 0.00 ± 0.01 | 0 | 2.26 ± 0.58 | | |
| 10 | 2.22 ± 1.19 | 0.02 ± 0.03 | 0.9 | 3.26 ± 0.91 | | |
| 15 | 2.63 ± 1.22 | 0.04 ± 0.05 | 1.5 | 3.50 ± 0.77 | | |
| 20 | 2.74 ± 1.19 | 0.08 ± 0.08 | 2.9 | 3.69 ± 0.84 | 0.06 | |
| 25 | 2.84 ± 1.20 | 0.12 ± 0.07 | 4.2 | 3.79 ± 0.86 | 0.07 | |
| 30 | 2.59 ± 0.92 | 0.14 ± 0.07 | 5.4 | 3.81 ± 0.97 | 0.22 ± 0.19 | 0.38 |
| 40 | 2.94 ± 1.12 | 0.20 ± 0.09 | 6.8 | 3.75 ± 0.87 | 0.32 ± 0.17 | 0.28 ± 0.10 |
| 50 | 2.72 ± 1.06 | 0.23 ± 0.11 | 8.5 | 4.05 ± 1.07 | 0.30 ± 0.19 | 0.33 ± 0.08 |
| 60 | 2.51 ± 1.17 | 0.25 ± 0.10 | 10.0 | 3.62 ± 1.02 | 0.34 ± 0.18 | 0.31 ± 0.05 |
| 75 | 2.18 ± 0.85 | 0.27 ± 0.10 | 12.4 | 3.37 ± 1.09 | 0.39 ± 0.21 | 0.44 ± 0.10 |
| 90 | 1.94 ± 0.81 | 0.28 ± 0.10 | 14.4 | 3.29 ± 0.94 | 0.43 ± 0.23 | 0.53 ± 0.53 |
| 105 | 1.80 ± 0.62 | 0.28 ± 0.11 | 15.6 | 3.10 ± 1.03 | 0.44 ± 0.24 | 0.47 ± 0.29 |
| 120 | 1.86 ± 1.15 | 0.29 ± 0.11 | 15.6 | 3.15 ± 1.05 | 0.51 ± 0.22 | 0.52 ± 0.33 |
| 135 | 1.77 ± 1.00 | 0.26 ± 0.07 | 14.7 | 2.90 ± 1.07 | 0.48 ± 0.21 | 0.53 ± 0.33 |
| 150 | 1.62 ± 0.87 | 0.27 ± 0.10 | 16.7 | 3.00 ± 1.18 | 0.52 ± 0.24 | 0.53 ± 0.33 |
| 165 | 1.54 ± 0.89 | 0.27 ± 0.09 | 17.5 | 2.70 ± 1.06 | 0.45 ± 0.22 | 0.47 ± 0.07 |
| 180 | 1.44 ± 0.87 | 0.25 ± 0.08 | 17.4 | 2.55 ± 1.02 | 0.40 ± 0.23 | 0.51 ± 0.23 |
| | | | | %** | 15.7 | 20.0 |

* Plasma concentration MEGX/lidocaine x 100%. Limit of quantitation 0.02 μg $ml^{-1}$.
** Plasma concentration metabolite/mepivacaine x 100%.

**TABLE 3**
**Comparison of the Pharmacokinetic Parameters (Mean ± s.d.) of Lidocaine and Mepivacaine after Axillary Administration of 40 ml 1.5% = 600 mg (+ 5 μg $ml^{-1}$ Adrenaline) in 15 Patients**

| Parameter | | *Lidocaine* | *Mepivacaine* | *p* |
|---|---|---|---|---|
| Subjects | M/F | 10/5 | 7/8 | |
| Body weight | kg | 80.4 ± 11.7 | 72.8 ± 13.9 | 0.12 |
| Age | Years | 55.5 ± 14.2 | 49.7 ± 12.3 | 0.24 |
| Length | cm | 173.5 ± 64.2 | 172.5 ± 7.6 | 0.70 |
| Dose | mg | 600 | 600 | |
| | mMol | 2.561 | 2.436 | |
| $AUC_{\infty}$ | mg h $l^{-1}$ | 10.5 ± 5.24 | 30.4 ± 22.6 | 0.0008 |
| $AUC_{3h}$ | mg h $l^{-1}$ | 5.48 ± 1.71 | 9.51 ± 2.59 | 0.0016 |
| $Cl_{\infty}$ | l $h^{-1}$ | 67.9 ± 28.9 | 26.9 ± 10.6 | 0.0008 |
| $Cl_{3h}$ | l $h^{-1}$ | 120 ± 39.8 | 66.6 ± 14.4 | 0.0016 |
| $V_d$ | l | 92.2 ± 61.2 | | |
| $V_{ss}$ | l | 229. ± 70.6 | | |
| $V_{\beta}$ | l | 241. ± 75.9 | 150.4 ± 27.12 | 0.0071 |
| $t_{1/2absorption}$ | h | 0.14 ± 0.05 | 0.064 ± 0.035 | 0.0022 |
| $t_{1/2\alpha}$ | min | 9.95 ± 14.3 | | |
| $t_{1/2\beta}$ | h | 2.86 ± 1.55 | 4.78 ± 2.38 | 0.0106 |
| MRT | h | 4.02 ± 1.79 | 7.00 ± 3.45 | 0.0087 |
| $t_{max}$ | h | 0.43 ± 0.19 | 0.41 ± 0.19 | 0.3225 |
| $C_{max}$ | mg $l^{-1}$ | 2.87 ± 1.19 | 3.89 ± 0.83 | 0.0157 |

p = Mann-Whitney two-tailed test.

**TABLE 4**
**Comparison of the Pharmacokinetic Parameters (Mean ± s.d.) of the Lidocaine and Mepivacaine Metabolites MEGX, 4-OH, and PPX after Axillary Administration of 40 ml 1.5% = 600 mg (+ 5 mg ml$^{-1}$ Adrenaline) in 15 Patients**

| Parameter | | *Lidocaine MEGX* | *p* | *Mepivacaine PPX* | *p* | *4-OH* | *p** |
|---|---|---|---|---|---|---|---|
| Subjects | M/F | 10/5 | | 7/8 | | | |
| Body weight | kg | 80.4 ± 11.7 | | 72.8 ± 13.9 | 0.12 | | |
| Age | Years | 55.5 ± 14.2 | | 49.7 ± 12.3 | 0.24 | | |
| Length | cm | 173.5 ± 64.2 | | 172.5 ± 7.6 | 0.70 | | |
| Dose | mg | 600 | | 600 | | | |
| | mMol | 2.561 | | 2.436 | | | |
| $AUC_{¥}$ | mg h $l^{-1}$ | 2.22 ± 1.10 | 0.22 | 67.1 ± 88.6 | 0.045 | 4.25 ± 2.56 | 0.73 |
| $AUC_{3h}$ | mg h $l^{-1}$ | 0.67 ± 0.24 | 0.22 | 1.11 ± 0.51 | 0.045 | 1.05 ± 0.052 | 0.74 |
| % AUCparent | | 13.0 | | 3.89 | | 3.24 | |
| $t_{1/2absorption}$ | h | 0.70 ± 0.43 | 0.17 | 1.20 ± 0.87 | 0.79 | 0.79 ± 0.75 | 0.30 |
| $t_{1/2ß}$ | h | 2.36 ± 2.35 | 0.73 | 1.48 ± 0.74 | 0.0628 | 6.48 ± 6.57 | 0.087 |
| MRT | h | 5.32 ± 3.22 | >0.8 | 4.90 ± 0.17 | 0.12 | 10.7 ± 8.90 | 0.29 |
| $t_{max}$ | h | 2.31 ± 0.84 | 0.0171 | 3.19 ± 0.28 | 0.16 | 1.86 ± 0.50 | **0.0052** |
| $C_{max}$ | mg $l^{-1}$ | 0.32 ± 0.12 | 0.0219 | 0.56 ± 0.30 | 0.18 | 0.45 ± 0.25 | 0.32 |

p = Mann-Whitney two-tailed test, between MEGX and 4-OH and PPX.

p *= Mann-Whitney two-tailed test, between 4-OH and PPX.

%AUCparent = AUCmetabolite (mMol h $l^{-1}$; 3 h)/AUC parent (mMol h $l^{-1}$; 3 h)

The volume of distribution ($V_b$) of mepivacaine is smaller than that of lidocaine (*p* = 0.0071), its $C_{max}$ value is higher, 3.89 ± 0.83 vs. 2.87 ± 1.19 mg $l^{-1}$, resp (*p* = 0.0157).

Table 4 shows the pharmacokinetic parameters of the metabolites. The $t_{max}$ and $C_{max}$ of the two xylidide metabolites differed significantly; with PPX the $C_{max}$ occurs later, and it is higher than the $C_{max}$ of MEGX.

### DISCUSSION

For axillary brachial plexus block a rapid onset of good surgical anaesthesia seems to be important, but above all the local anaesthetic agent should be safe. (±)Mepivacaine and lidocaine both fulfill these criteria as reported by several authors[5,7,11,16,17,18]. Eriksson reported that with lidocaine (450 mg without adrenaline) 6 out of 20 patients showed signs of CNS toxicity about 10–15 min after axillary administration, i.e., pronounced dizziness and nearly a loss of consciousness in one patient. Reported onset time for lidocaine was 26.6 ± 4.1 min.[18]. We added adrenaline to slow absorption and to reduce systemic side effects.

The onset of motor block by lidocaine and mepivacaine with 5 µg ml$^{-1}$ adrenaline was similar. Development of motor blockade in the present study showed a wide range of rates and proceeds apparently independently of the plasma concentration time curve (Fig. 1 A,B and Fig. 4). The end-point of 100% motor blockade was achieved within 2 min by four patients in the lidocaine group and by five patients in the mepivacaine group, while in each group one patient needed a full 20 min. Meanwhile, the local anaesthetic is absorbed and appears in the general circulation.

The absorption of both lidocaine and mepivacaine from the axilla region was relatively fast ($t_{max}$ 0.43 h, **respectively** 0.41 h). The maximum plasma concentrations of both lidocaine and mepivacaine were in agreement with those reported earlier[18,19,20,21,22,23] and stayed well below the toxic concentrations of 5–10 µg ml$^{-1}$[24,25,26]. Thereafter, lidocaine was eliminated according a

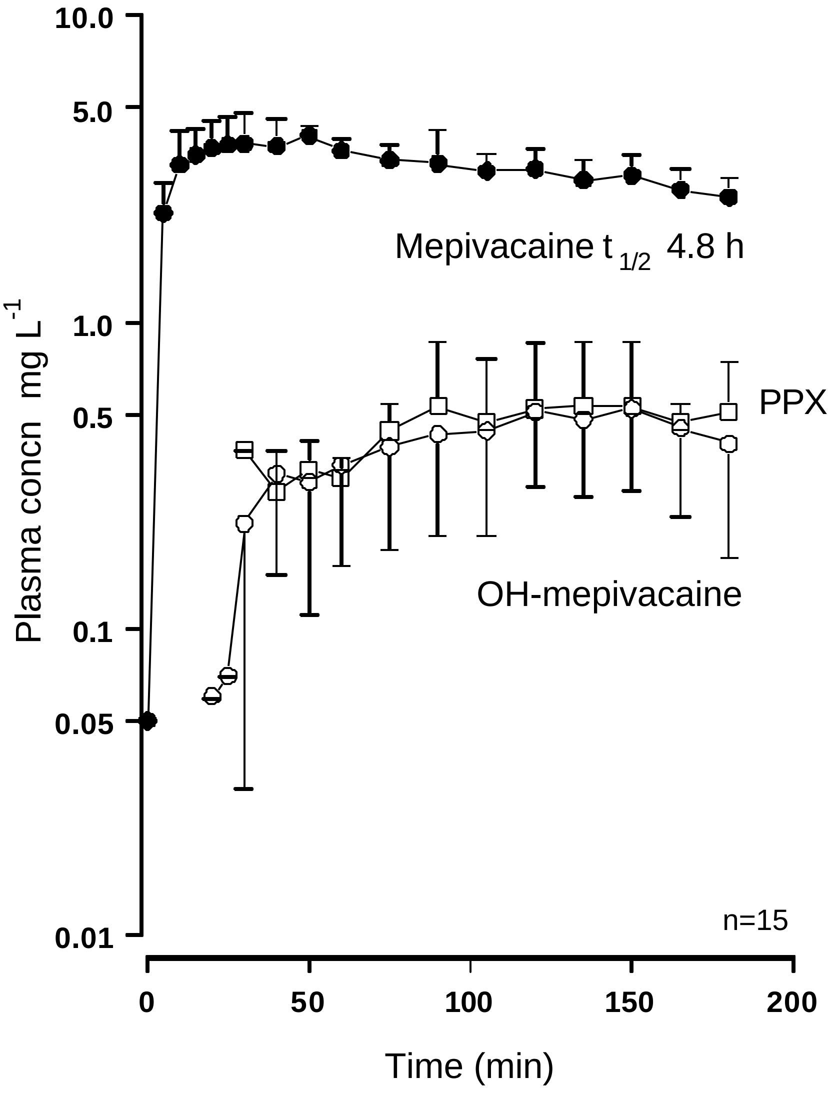

**FIGURE 3.** Mean plasma concentration–time curves of mepivacaine (mg $l^{-1}$ ± s.d.), and its metabolites 4´-hydroxymepivacaine (4-OH, open dots) and 2,6-pipecoloxylidide (PPX, open squares) after axillary administration of 600 mg mepivacaine (+ 5 mg $ml^{-1}$ adrenaline) (n = 15).

biexponential decay with $t_{1/2\alpha}$ of 9.95 ± 14.3 min and a $t_{1/2\beta}$ of 2.86 ± 1.55 h. Mepivacaine was eliminated according to a monexponential decay with a $t_{1/2\beta}$ of 4.78 ± 2.38 h, which differed from that of lidocaine ($p$ = 0.0106). The pharmacokinetic data shown in Table 3 correspond with those previously reported[6,7,10,11].

The large %CV in the $t_{1/2\alpha}$ of lidocaine was caused by some exceptionally long $t_{1/2\alpha}$ values. In the lidocaine group 6 out of 15 patients showed a long $t_{1/2\alpha}$ of 23.9 ± 13.5 min, which differed from the short values of 0.63 ± 0.71 min ($p$ = 0.0018). Three patients did not develop a sensory block in all nerves after lidocaine injection; this may be due to poor distribution over the nerves. However, this lack of sensory block could not be related to the individual plasma concentration-time curves and pharmacokinetics. The differences in the mepivacaine group were much smaller.

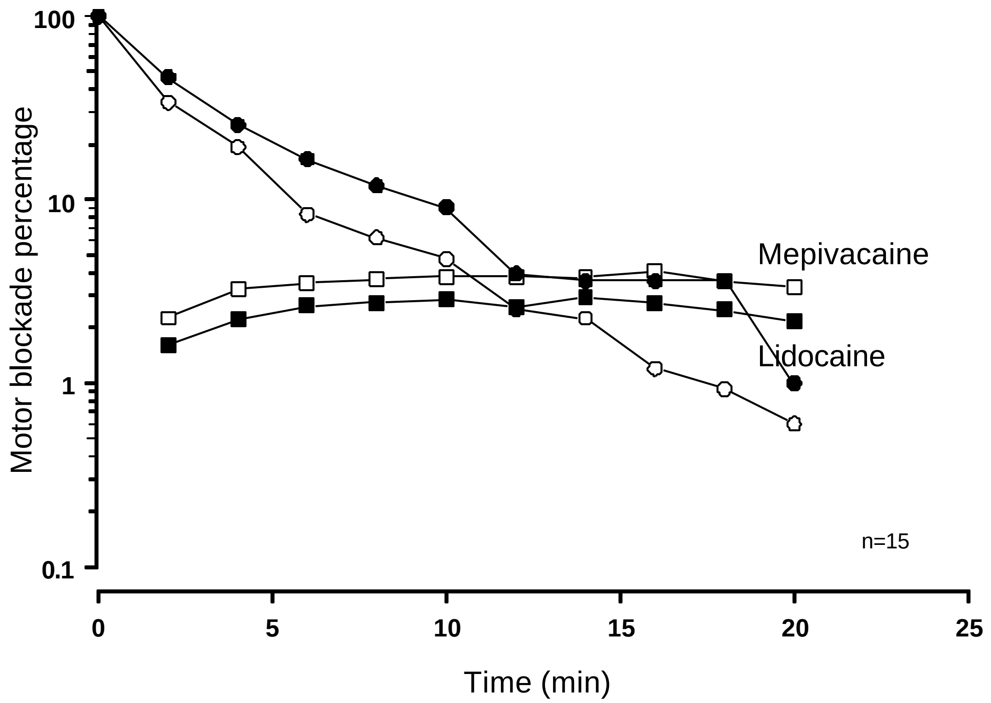

**FIGURE 4**. Mean percentage motor blockade (dots) and mean plasma concentration–time curves (squares) of lidocaine (solid symbols), and mepivacaine (open symbols) after axillary administration of 600 mg (+ 5 μg $ml^{-1}$ adrenaline) (n = 15). Decrease in motor blockade can be tested only before surgery, while testing of the recovery is not possible due to appendages.

### $AUC_\infty$ vs. $AUC_{3h}$

The difference between the pharmacokinetics of lidocaine and mepivacaine can be attributed solely to the difference in $C_{max}$ and $t_{1/2}$ values, which affects the AUC∞, and $AUC_t$ and thus the calculated total body clearance. Clearance values were based on CL = F.Dose/$AUC_{3h}$ with the assumption that F = 1. The clearance values of the metabolite (MEGX, PPX, 4-OH) must be multiplied by the fraction of the dose that is converted into metabolite. The plasma concentration of both parent compounds did not reach the limit of quantification at t = 3 h, which makes the difference between the extrapolated $AUC_\infty$ and $AUC_{3h}$ constant. The $AUC_{3h}$ is 52% of the $AUC_\infty$ for lidocaine and 31% for mepivacaine. The $AUC_{3h}$ is required for the calculation of the AUC of the metabolite (MEGX, PPX, 4-OH) because after 3 h sampling this compound shows a plateau plasma concentration. Elimination must be visible when a much longer sampling period could have been applied. This was impossible by the nature of the type of day case surgery and the allotted time by the hospital rules.

Calculated clearance was based on the assumption of 100% bioavailability (F = 1). The total body clearances based on $AUC_\infty$ and $AUC_{3h}$ of lidocaine equaled the maximum average liver blood flow of 1.5 l $min^{-1}$ (90 l $h^{-1}$), while that of mepivacaine was half that of the liver blood flow.

### Comparison of Metabolism

The metabolism of lidocaine and mepivacaine proceeds via cytochrome P450 isoenzymes, resulting in N-hydroxylation, N-dealkylation (MEGX, GX), and the principal reaction of 4-hydroxylation

(80% of the dose in urine)[27,28,29,30,31,32,33,34,35,36,37,38]. In this study we were able to measure the plasma concentration of MEGX, which increased to 17% of the lidocaine plasma concentration, and its $AUC_t$ was 13% of that of the $AUC_t$ of parent drug. The plasma concentrations of the mepivacaine metabolites increased to 15.7% (4-OHmepivacaine) and 20.0% (PPX) of the concentration of the parent drug. The $AUC_t$ of the mepivacaine metabolites was 3.89% (PPX) and 3.24% (4-OHmepivacaine) of the $AUC_t$ of mepivacaine. Measurement of the metabolite MEGX was not possible when lidocaine was administered in IVRA at a (low) dose of 200 mg[39].

The enantiomers of (±)mepivacaine show the same pharmacokinetic behaviour, which is also similar to the pharmacokinetics of the racemate[7,40], while D(–)-mepivacaine is more active than L(+)-mepivacaine[41,42,43].

### CONCLUSION

It can be concluded that both lidocaine and mepivacaine are suitable and safe agents for axillary brachial plexus block with rapid onset of good surgical analgesia. This is in line with everyday practice in most institutions. Both compounds are absorbed rapidly from the neurovascular sheath and eliminated with a short $t_{1/2ß}$ of 2.9 h for lidocaine and 4.8 h for mepivacaine.

Mepivacaine is a racemic mixture, which to date is considered to be not theoretically correct, however clinically this racemic mixture is effective and nontoxic in the dose applied. Although there are kinetic differences between the two drugs, they have no clinical consequences.

### ACKNOWLEDGEMENT

This study was funded by the Foundation for Development of Anaesthesiology, Nijmegen, the Netherlands.
